# 964 A Qualitative Assessment of Prehospital Burn Care in a Metropolitan Area

**DOI:** 10.1093/jbcr/iraf019.495

**Published:** 2025-04-01

**Authors:** Ronnie Adams, Steve Elmgren, Lauren Nosanov, Laura Johnson, Yuk Ming Liu

**Affiliations:** Grady Burn Center; Grady Hospital; Emory University School of Medicine; Walter L. Ingram Burn Center at Grady Memorial Hospital; Emory University - Walter L. Ingram Burn Center

## Abstract

**Introduction:**

Burns are among the most common traumatic injuries and are associated with significant morbidity and mortality; however, many prehospital providers remain unfamiliar with current management algorithms due to infrequent exposure. Prehospital burn management encompasses wound care, fluid resuscitation, and pain management, all of which must be accomplished while ensuring prompt transportation to a Burn Center. Proper resuscitation is crucial to clinical outcomes, but care guidelines vary widely by various Emergency Medical Services (EMS). We sought to characterize and evaluate the burn care provided by EMS agencies in our metropolitan region to identify opportunities for improvement and education.

**Methods:**

Surveys assessing clinical experience, burn-specific education, and provider comfort were distributed at American Burn Association (ABA) Advanced Burn Life Support (ABLS) courses and EMS conferences, and were sent to EMS leadership to circulate to staff. EMS clinical protocols were also obtained, and those guidelines were compared to the ABLS course manual. Survey design and analysis were conducted with Qualtrics.

**Results:**

38 survey responses from providers representing 19 EMS agencies, spanning fire-based, private, and helicopter EMS, as well as 8 protocols were obtained and qualitatively analyzed. Respondents were primarily paramedics (61%), and 34 (89%) had renewed their license in the past two years. Notably, 35/38 (92%) stated they transported five or fewer burn patients within the past year, and 28/35 (80%) stated they were not comfortable establishing vascular access through a burn wound. Continuing education was most commonly obtained through formal courses (66%), podcasts (42%), and departmental training (34%). Qualitative review of the protocols revealed varied formulas for fluid resuscitation, including, Parkland, Consensus, Rule of 10s, and using systolic blood pressure as a guide. Approved pain medications included morphine, ketamine, and fentanyl; two agencies mentioned hydroxocobalamin in their protocol. Thermoregulation measures were mentioned in three protocols. Of note, two protocols were updated to align with ABA guidelines during the survey period due to a concurrent quality initiative.

**Conclusions:**

Prehospital providers lack high-volume exposure to this patient population and exhibit low comfort levels. Minimal information on burns is included in regular continuing education. Practices differ between EMS services, despite the fact that the quality of prehospital care has a sizeable impact on outcomes.

**Applicability of Research to Practice:**

Continued efforts to align protocols with ABA guidance and expand ABLS courses will ensure consistent, quality burn care in the prehospital setting.

**Funding for the Study:**

N/A

